# Genotype B of *Killer Cell Immunoglobulin-Like Receptor* is Related with Gastric Cancer Lesions

**DOI:** 10.1038/s41598-018-24464-2

**Published:** 2018-04-17

**Authors:** Eric G. Hernandez, Oswaldo Partida-Rodriguez, Margarita Camorlinga-Ponce, Miriam Nieves-Ramirez, Irma Ramos-Vega, Javier Torres, Martha Perez-Rodriguez

**Affiliations:** 1 Unidad de Investigación Médica en Inmunología, Hospital de Pediatría Centro Médico Nacional Siglo XXI, Instituto Mexicano del Seguro Social, Cuauhtémoc 330, Col. Doctores, CP 06720, Ciudad de México, Mexico; 2 Unidad de Investigación Médica en Enfermedades Infecciosas, Hospital de Pediatría Centro Médico Nacional Siglo XXI, Instituto Mexicano del Seguro Social, Cuauhtémoc 330, Col. Doctores, CP 06720, Ciudad de México, Mexico

## Abstract

NK cells are important in innate immunity for their capacity to kill infected or cancer cells. The killer cell immunoglobulin-like receptors (KIR) are a family of polymorphic genes with inhibitory and activating functions. The main driving force for gastric cancer (GC) development is a chronic response, which causes an increase of NK cells in the gastric mucosa. The aim of this work was to study polymorphisms in KIR genes in patients with either GC or non-atrophic gastritis (NAG). We studied 242 patients (130 with NAG and 112 with GC) and contrasted with 146 asymptomatic individuals. We analyzed diversity in the content and localization of KIR genes in the different clinical groups studied. Four activating and one inhibitory genes were associated with GC: 2DS1 (OR 3.41), 2DS3 (OR 4.66), 2DS5 (OR 2.25), 3DS1 (OR 3.35) and 2DL5 (OR 3.6). The following were also found as risk factors for GC: Bx genotype (OR 4.2), Bx-Bx centromere-telomere (OR 2.55), cA01|cB03 (OR 36.39) and tB01|tB01 (OR 7.55) gene content and three B motifs (OR 10.9). Polymorphisms in KIR genes were associated with GC and suggest that mutated NK cells may contribute to GC development by increasing gastric mucosa inflammation, leading to constant tissue damage.

## Introduction

NK cells represent a subset of lymphoid cells that are components of innate immunity acting as first line of defense against viral infection and other pathogens, and in the early cellular transformation and tumor surveillance^1^. The functions of NK cells are partly regulated by the family of KIR receptors (killer cell immunoglobulin-like receptor) coded by 11 genes (*2DL1*, *2DL2/2DL3*, *2DL4*, *2DL5*, *2DS1*, *2DS2*, *2DS4*, *2DS3/2DS5*, *3DL1/3DS1*, *3DL2 and 3DL3*) and two pseudogenes (*2DP1* and *3DP1*) located on the chromosome 19q13.4^2–4^. Some of these genes may present sequence variations; thus, it has been reported a 22 bp deletion in the second extracellular domain of *2DS4* that affect substantially the sequence of amino acids, whereas the exon 2 can be absent in *3DP1*^5^. Also, it has been found that *2DL5* gene is encoded by different loci (A and B)^6^. The KIR family is primarily expressed on NK cells, but they can also be expressed on CD4, CD8 and γδ T cells^7–9^. There are four promoter types based on intermediate promoters (ProI), which are associated with distinct expression in KIR genes, thus *2DL2*, *2DS2 and 2DL3* are the first to be expressed by NK cells after Hemopoietic Stem Cell Transplantation^10^. The *3DL3* is not expressed by circulating CD56 dim NK cells, and *2DL4* is expressed by CD56-bright and dim NK cells in a non-variegated manner; and finally, the remaining KIR genes are expressed by CD56-dim NK cells^10,11^. T cells express *3DL2* more than other KIR genes, probably as a result of ProI activation earlier in the development of T cell^10^. In addition, the KIR gene family has bi-directional promoters, which control variegated expression, whereas ProI correlates with protein expression^10^.

**Figure 1:**
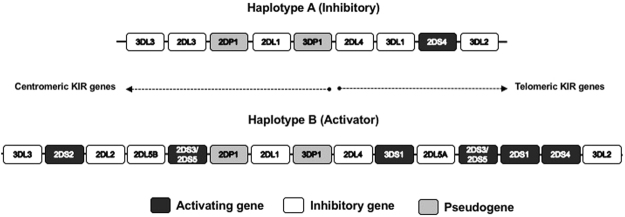
Composition of KIR haplotypes (**A** and **B**) based on gene content. KIR genes are tightly organised head-to-tail over approximately 150 kb within the Leukocyte Receptor Complex (LCR). Inhibitory KIR genes are shown in white, activating genes in black and pseudogenes in gray.

Composition of KIRs may be complex, thus, two haplotypes (A and B) and genotypes (AA and Bx, where x can be A or B) have been reported for KIR based on gene content^12^ (Fig. 1). Actually, there are over 500 different Bx genotypes (http://www.allelefrequencies.net). KIR genotype AA is homozygous for the A haplotype, which is an inhibitory haplotype formed by the loci *3DL3*, *2DL3*, *2DP1*, *2DL1*, *3DP1*, *2DL4*, *3DL1*, *2DS4* and *3DL*2; whereas Bx genotype has either one (AB heterozygous) or two (BB homozygous) B haplotypes, and is an activator haplotype (formed by *3DL3*, *2DS2*, *2DL2*, *2DL5B*, *2DS3/2DS5*, *2DP1*, *2DL1*, *3DP1*, *2DL4*, *3DS1*, *2DL5A*, *2DS3/2DS5*, *2DS1*, *2DS4* and *3DL2* genes)^13^. The A haplotype usually has a fixed number of genes, while B has a variable gene content with additional activating KIR genes. KIR haplotypes consists of two regions, the centromeric region from *3DL3* to *3DP1*, and the telomeric region from *2DL4* to *3DL2;* and both regions can be cenA or cenB, and telA or telB depending on the haplotype^13,14^. *2DL5*, *2DS3* and *2DS5* genes have been identified in centromeric and/or telomeric region^14^. Based on the gene content 9 centromeric regions (cA01, cA02, cA03, cB01, cB02, cB03, cB04, cB05 and cB06) and 8 telomeric regions (tA01, tB01, tB02, tB03, tB04, tB05, tB06 and tB07) have been described^14–17^. KIR B haplotype can also be classified according to B content genes, and B content score is calculated by adding the number of cenB and/or telB motifs in each genotype^18^.

*Helicobacter pylori* (*H*. *pylori*) infects the gastric mucosa of over 50% of the world population and represents the main risk factor to develop gastric cancer (GC) and duodenal ulcer^19,20^. Different immune cells are involved in the development of gastric pathologies by causing a chronic, unregulated mucosal inflammation^21^. Thus, cells of the innate and adaptive system such as T lymphocytes and natural killer (NK) cells have a critical role in the regulation of the immune response^21^. *H*. *pylori* causes an increase of NK cells in the gastric mucosa, where they produce TNF-α and INF-γ^21–23^, and have an important role in the inflammatory process that drive tissue damage. In this work we aimed to study polymorphisms in KIR receptors genes and identify any possible association with GC.

## Results

The characteristics of the population studied are described in Table 1. We studied two groups of patients, one with a diagnosis of NAG and the other with GC, formed by 130 and 112 patients respectively, and both were compared with an asymptomatic group (n = 146). It can be observed that the NAG group showed significantly higher seroprevalence to *H*. *pylori* and to CagA, with an OR of 3.23 and 2.74, respectively, as compared to the asymptomatic patients.

**Table 1:** Characteristics of the groups of patients studied.

Diagnosis group	n^a^	Age, years, median (IQR)^b^	Female/Male	*H*. *pylori* +/− (%)	OR (95% CI)^c^	CagA + /− (%)	OR (95% CI)^c^
Asymptomatic	146	41 (36–50)	54/92	109/37 (74.7)		74/72 (50.7)	
Non-atrophic gastritis	130	47 (39–57)	93/37	111/19 (85.4)^d^	3.23 (1.39–7.54)	89/41 (68.5)^d^	2.74 (1.39–5.42)
Gastric cancer	112	59 (49–72)	49/63	87/25 (77.7)	2.56 (0.93–7.02)	65/47 (58)	2.27 (0.99–5.21)

^a^n = number of subjects. ^b^IQR = interquartile range. ^c^OR estimated using the asymptomatic group as the reference and adjusted by age and gender. ^d^*p* < 0.05.

### KIR genes

In order to characterize the KIR genotype frequencies in the study groups, genomic DNA was isolated from peripheral blood leukocytes, and the KIR genes responsible for the activating signals (*2DS1*, *2DS2*, *2DS3*, *2DS4*, *2DS5*, *3DS1*), the inhibitory signals (*2DL1*, *2DL2*, *2DL3*, *2DL4*, *2DL5*, *3DL1*, *3DL2*, *3DL3*), and the two pseudogenes (*2DP1* and *3DP1*) were genotyped using single specific primer-polymerase chain reaction (SSP-PCR). KIR genotypes were assembled according to the presence or absence of each gene locus.

The frequency data obtained was analyzed between groups to determine differences in KIR genes between asymptomatic and disease groups. The framework genes of centromeric (*3DL3* and *3DP1*) and telomeric (*2DL4* and *3DL2*) regions were present in 100% of the three groups studied. The frequencies of *2DL1* (99.3%, 99.2% and 100%), *2DL3* (97.9%, 96.9% and 92.9%), *2DS4* (93.8%, 100% and 88.4%) and *2DP1* (99.3%, 99.2% and 100%) genes were not statistically different among the groups (Asymptomatic, NAG and GC, respectively). The KIR genes with a significant association with disease are shown in Table 2. When compared with healthy controls, most of the activating and inhibitory genes studied were found significantly associated with either NAG or GC, whereas some showed an increasing tendency of association from NAG to GC, like *2DS1* (OR of 2.56 to OR 5.45), *3DS1* (OR of 3.52 to OR 4.75) and *2DL5* (OR of 3.77 to OR 6.21). In contrast, *2DS3* presented a significantly decreasing tendency of association from NAG to GC, (OR of 187.7 to OR 24.98). *2DS2* and *2DL2* showed significant association with NAG (OR of 3.75 for the two genes), whereas *3DL1* was associated with protection for GC (OR 0.61). The above associations remained significant in a multivariate model of analyses (Table 3), where it can be observed that age was constantly associated with risk for GC, and *H*. *pylori* and gender for NAG. The multivariate analyses showed that *2DS1*, *2DS3*, *2DS5*, *3DS1* and *2DL5* were found as risk for GC, whereas *3DL1* was found as protective (OR 0.21) (Table 3).

**Table 2:** Distribution of KIR genes giving significant differences between clinical groups.

Genes	Asymptomatics	Non-atrophic gastritis		Gastric cancer	
	n = 146^a^ (%)	n = 130^a^ (%)	OR (95% CI)^b^	n = 112^a^ (%)	OR (95% CI)^b^
Activating					
2DS1^d^	56 (38.4)	72 (55.4)	2.56 (1.35–4.85)	79 (70.5)	5.45 (2.20–13.54)
2DS2	48 (32.9)	70 (53.8)	3.75 (1.87–7.52)	35 (31.3)	NS^c^
2DS3^d^	21 (14.4)	113 (86.9)	187.78 (43.83–804.46)	50 (44.6)	24.98 (5.85–106.61)
2DS5	46 (31.5)	18 (13.8)	0.20 (0.081–0.48)	55 (49.1)	3.16 (1.31–7.58)
3DS1^d^	55 (37.7)	76 (58.5)	3.52 (1.80–6.86)	76 (67.9)	4.75 (1.97–11.50)
Inhibitory					
2DL2	48 (32.9)	70 (53.8)	3.75 (1.87–7.52)	36 (32.1)	NS^c^
2DL5^d^	62 (42.5)	86 (66.2)	3.77 (1.92–7.38)	81 (72.3)	6.21 (2.41–15.98)
3DL1	139 (95.2)	130 (100.0)	NS^c^	99 (88.4)	0.61 (0.008–0.45)

^a^n = number of subjects. ^b^Comparisons were made using the asymptomatic group as the reference group, *pc* < 0.05 and OR (95% C.I.) were adjusted by age and gender. ^c^NS = not significant. 2DS2 = 1.13 (0.46–2.72), 2DL2 = 1.13 (0.46–2.72. ^d^Linear trend analysis for 2DS1, 3DS1 and 2DL5 genes from asymptomatic to non-atrophic gastritis to gastric cancer (P < 0.00001).

**Table 3:** Multivariate logistic regression analysis of KIR genes associated with gastric disease.

Gene	Variable	Non-atrophic gastritis		Gastric cancer	
		*p*	OR (95% CI)^a^	*P*	OR (95% CI)^a^
2DS1	2DS1^b^	0.005	2.14 (1.25–3.65)	<0.0001	3.41 (1.87–6.22)
	*H*. *pylori* +	0.038	2.07 (1.04–4.10)	NS ^c^	
	male	<0.0001	0.23 (0.13–0.39)	NS	
	≥50 years	0.013	2.01 (1.16–3.47)	<0.0001	8.09 (4.48–14.62)
2DS2	2DS2^b^	0.001	2.71 (1.57–4.67)	NS	
	*H*. *pylori* +	0.016	2.33 (1.17–4.64)		
	male	<0.0001	0.22 (0.13–0.38)		
	≥50 years	0.014	2.01 (1.15–3.51)		
2DS3	2DS3^b^	<0.0001	32.38 (15.9–65.7)	<0.0001	4.66 (2.34–9.26)
	*H*. *pylori* +	NS		NS	
	male	0.007	0.37 (0.20–0.80)	NS	
	≥50 years	NS		<0.0001	8.57 (4.70–15.66)
2DS5	2DS5^b^	0.006	0.40 (0.21–0.80)	0.008	2.25 (1.24–4.10)
	*H*. *pylori* +	0.014	2.32 (1.18–4.56)	NS	
	male	<0.0001	0.27 (0.16–0.46)	NS	
	≥50 years	0.009	2.08 (1.20–3.61)	<0.0001	8.90 (4.97–15.94)
3DS1	3DS1^b^	0.001	2.49 (1.45–4.27)	<0.0001	3.35 (1.83–6.13)
	*H*. *pylori* +	NS		NS	
	male	<0.0001	0.23 (0.13–0.39)	NS	
	≥50 years	0.011	2.04 (1.17–3.56)	<0.0001	8.37 (4.63–15.13)
2DL2	2DL2^b^	<0.0001	2.75 (1.60–4.76)	NS	
	*H*. *pylori* +	0.013	2.40 (1.20–4.40)		
	male	<0.0001	0.22 (0.13–0.38)		
	≥50 years	0.013	2.02 (1.16–3.53)		
2DL5	2DL5^b^	<0.0001	2.97 (1.72–5.12)	<0.0001	3.60 (1.94–6.67)
	*H*. *pylori* +	0.037	2.10 (1.04–4.18)	NS	
	male	<0.0001	0.23 (0.13–0.40)	NS	
	≥50 years	0.006	2.21 (1.26–3.87)	<0.0001	8.75 (4.82–15.91)
3DL1	3DL1^b^	NS		0.005	0.21 (0.07–0.63)
	*H*. *pylori* +			NS	
	male			NS	
	≥50 years			<0.0001	9.93 (5.46–18.05)

^a^Comparisons were made with the asymptomatic as the reference group. ^b^Adjusted by the other independent variables. ^c^NS = not significant.

### KIR haplotypes

In order to determine the association of genotypes with the disease groups, the genotypes were grouped as A or B based on gene content. We found that the genotype AA (inhibitory) was highly protective for NAG (OR 0.04, 95% CI 0.01–0.12) and GC (OR 0.22, 95% CI 0.08–0.57), whereas the genotype Bx (activator) was found as risk for both diseases (NAG OR 26.35, 95% CI 8.26–84.11; GC OR 4.57, 95% CI 1.74–12.0). Also, the results show that in our population the frequency of the genotype AA was close to 44%, while genotype Bx was 56%. The multivariate analyses confirmed the association with protection for genotype AA and with risk for genotype Bx (Table 4). As before, in this multivariate analyses gender was associated with NAG and age with GC.

**Table 4:** Multivariate logistic regression analysis of distribution of KIR genotype and variables in KIR centromere-telomere and its association with gastric disease.

Genotype	Variable	Non-atrophic gastritis		Gastric cancer	
		P	OR (95% CI)^a^	p	OR (95% CI)^a^
AA	AA^b^	<0.0001	0.073 (0.03–0.18)	<0.0001	0.23 (0.12–0.47)
	*H*. *pylori*	NS^c^		NS	
	male	<0.0001	0.22 (0.12–0.40)	NS	
	≥50 years	0.036	1.90 (1.04–3.45)	<0.0001	8.18 (4.52–14.91)
Bx	Bx^b^	<0.0001	13.62 (5.68–32.61)	<0.0001	4.2 (2.11–8.49)
	*H*. *pylori*	NS		NS	
	male	<0.0001	0.22 (0.12–0.40)	NS	
	≥50 years	0.036	1.90 (1.04–3.45)	<0.0001	8.18 (4.52–14.91)
**Centromere-Telomere**	**Variable**	**P**	**OR (95% CI)** ^**a**^	**p**	**OR (95% CI)** ^**a**^
cAcA-tAtA	cAcA-tAtA^b^	<0.0001	0.073 (0.03–0.18)	<0.0001	0.23 (0.12–0.47)
	*H*. *pylori*	NS^e^		NS	
	male	<0.0001	0.22 (0.12–0.40)	NS	
	≥50 years	0.036	1.90 (1.04–3.45)	<0.0001	8.18 (4.52–14.91)
cBx-tBx	cBx-tBx^b^	<0.0001	7.08 (3.80–13.17)	0.008	2.55 (1.27–5.10)
	*H*. *pylori*	0.027	2.28 (1.10–4.73)	NS	
	male	<0.0001	0.21 (0.11–0.37)	NS	
	≥50 years	NS		<0.0001	8.73 (4.88–15.61)

^a^Comparisons were made with the asymptomatic as the reference group. ^b^Adjusted by the other independent variables. ^c^NS = not significant. AA = homozygote to A. Bx = homozygote or heterozygote to B. c = centromere. t = telomere.

Because the centromeric and telomeric regions have different gene content, genotypes AA and Bx were analyzed based on their distribution. Four different combinations of centromeric-telomeric (cAcA-tAtA, cAcA-tBx, cBx-tAtA and cBx-tBx) region of the genotypes were found. The cAcA-tAtA distribution was found significantly associated with protection (NAG OR 0.04, 95% CI 0.01–0.12; GC OR 0.22, 95% CI 0.08–0.57) and cBx-tBx with risk (NAG OR 10.65, 95% CI 4.84–23.44; GC OR 11.15, 95% CI 3.33–37.30) for both, NAG and GC. We noted that cAcA-tAtA was constant in asymptomatic population (43.8%), whereas cBx-tBx was more frequent in NAG (56.2%, vs 16.4% in asymptomatic and 33% in GC) and cAcA-tBx in GC (42.9%, vs 22.6% in asymptomatics and 33.1% in NAG). The multivariate analyses confirmed the association with protection for cAcA-tA-tA and with risk for cBx-tBx (Table 4).

### KIR B score

The B content score analyses showed an association with protection of genotype AA for NAG (OR 0.04) and GC (OR 0.22) whereas the score 2 was associated with NAG (OR 9.7), and score 3 with GC (OR 78.7) (Table 5). The above associations were confirmed in a multivariate analysis (Table 6), and the risk of score 3 with GC remained as high (OR 10.9) as with age (OR 9.9).

**Table 5:** KIR B content score and its association with gastric disease.

Genotype	B score	Centromere-Telomere	Asymptomatics	Non-atrophic gastritis		Gastric cancer	
			n = 146^a^ (%)	n = 130^a^ (%)	OR (95% CI) ^b^	n = 112^a^ (%)	OR (95% CI)^b^
AA	0	cAcA-tAtA	64 (43.8)	7 (5.4)	0.04 (0.01–0.12)^c^	16 (14.3)	0.22 (0.08–0.57)^c^
Bx	1	cAcA-tAtB	54 (37)	50 (38.5)	NS^d^	56 (50)	NS
		cAcB-tAtA					
	2	cAcA-tBtB	25 (17.1)	71 (54.6)	9.7 (4.4–21.5)^c^	26 (23.2)	NS
		cAcB-tAtB					
		cBcB-tAtA					
	3	cAcB-tBtB	3 (2.1)	2 (1.5)	NS	14 (12.5)	78.7 (4.9–1246.6)^c^
		cBcB-tAtB					
	4	cBcB-tBtB	0	0		0	

^a^n = number of subjects; ^b^Comparisons were done using the asymptomatic group as the reference group and OR (95% C.I.) adjusted by age and gender. ^c^*pc* < 0.05. ^d^NS = not significant. AA = homozygote to A. Bx = homozygote or heterozygote to B. c = centromere. t = telomere. B score is the number of cB and/or tB motifs in each genotype^18^.

**Table 6:** Multivariate logistic regression analysis of KIR B content score and its association with gastric diseases.

B score	Variable	Non-atrophic gastritis		Gastric cancer	
		p	OR (95% CI)^a^	P	OR (95% CI)^a^
0	0^b^	<0.0001	0.073 (0.03–0.18)	<0.0001	0.23 (0.12–0.47)
	*H*. *pylori*	NS^c^		NS	
	male	<0.0001	0.22 (0.12–0.40)	NS	
	≥50 years	0.036	1.90 (1.04–3.45)	<0.0001	8.18 (4.52–14.91)
2	2^b^	<0.0001	6.6 (3.5–12.2)		
	*H*. *pylori*	0.013	2.5 (1.2–5.1)		
	male	<0.0001	0.21 (0.12–0.38)		
	≥50 years	0.042	1.8 (1.02–3.3)		
3	3^b^			0.001	10.9 (2.7–43.8)
	*H*. *pylori*			NS	
	male			NS	
	≥50 years			<0.0001	9.9 (5.4–17.9)

^a^Comparisons were made with the asymptomatic as the reference group. ^b^Adjusted by the other independent variables. ^c^NS = not significant.

### KIR centromeric and telomeric distribucion

To further analyze the association of gene content of centromeric or telomeric regions with NAG and GC we studied the different combinations that have been reported. The multivariate analyses showed a strong association of risk for GC with cA01|cB03 (OR 36.3) and tB01|tB01 (OR 7.55), and of protection with tA01|tA01 (OR 0.23) (Table 7). The analysis also showed a strong risk for NAG with tA01|tB0X (OR 26.04) and high protection with tA01|tA01 (OR 0.08).

**Table 7:** Multivariate logistic regression analysis of distribution of KIR according to centromeric and telomeric gene content.

Gene content	Variable	Non-atrophic gastritis		Gastric cancer	
		*p*	OR (95% CI)^a^	*p*	OR (95% CI)^a^
cA01|cA01	cA01|cA01^b^	<0.0001	0.28 (0.16–0.48)	NS^c^	
	*H*. *pylori*	0.02	2.30 (1.14–4.62)		
	male	<0.0001	0.21 (0.12–0.38)		
	≥50 years	0.014	2.04 (1.16–3.59)		
cA01|cB02	cA01|cB02^b^	<0.0001	2.83 (1.59–5.03)	NS	
	*H*. *pylori*	0.024	2.20 (1.11–4.38)		
	male	<0.0001	0.21 (0.13–0.38)		
	≥50 years	0.016	1.98 (1.14–3.46)		
cA01|cB03	cA01|cB03^b^	NS		0.001	36.39 (4.32–306.85)
	*H*. *pylori*			NS	
	male			NS	
	≥50 years			<0.0001	10.65 (5.80–19.55)
tA01|tA01	tA01|tA01 ^b^	<0.0001	0.08 (0.04–0.16)	<0.0001	0.23 (0.12–0.43)
	*H*. *pylori*	NS		NS	
	male	<0.0001	0.23 (0.13–0.43)	NS	
	≥50 years	NS		<0.0001	7.88 (4.32–14.36)
tA01|tB01	tA01|tB01^b^	0.001	2.51 (1.45–4.35)	0.016	2.06 (1.15–3.70)
	*H*. *pylori*	NS		NS	
	male	<0.0001	0.23 (0.14–0.40)	NS	
	≥50 years	0.013	2.02 (1.16–3.50)	<0.0001	7.90 (4.44–14.07)
tA01|tB0X	tA01|tB0X^b^	<0.0001	26.04 (7.45–90.97)	NS	
	*H*. *pylori*	0.006	3.0 (1.38–6.54)		
	male	<0.0001	0.30 (0.17–0.54)		
	≥50 years	NS			
tB01|tB01	tB01|tB01^b^	NS		0.001	7.55 (2.31–24.70)
	*H*. *pylori*			NS	
	male			NS	
	≥50 years			<0.0001	10.45 (5.70–19.16)

^a^Comparisons were made with the asymptomatic as the reference group. ^b^Adjusted by the other independent variables. ^c^NS = not significant. c = centromere. t = telomere. The number was according gene content^15–17^. 0X = the number had not been assigned so far.

## Discussion

In the present study, we showed the association of KIR receptors family genes with gastric pathologies. This association was observed with presence of genes, with genotypes, with centromere-telomere regions and with B score. Of particular interest, we observed that both activator and inhibitor genes were associated with GC. A balance between activator and inhibitor genes is necessary during immune surveillance by NK receptors; but when unregulated, their activity may contribute to pathogenesis of diverse diseases including tumor development^24^. We found that the presence of *2DS1*, *2DS3* and *3DS1* activating genes was associated with risk for both, NAG and GC. In studies in other populations *2DS1* has also been found associated with cancer, although with breast cancer^25^, whereas *2DS1*, *2DS3* and *3DS1* have been associated with more severe pulmonary tuberculosis in Asian population^26^; *2DS1* and *3DS1* with ankylosing spondylitis in Spain^27^, and *2DS2* with rheumatoid arthritis in Latin America^28^. In our population the activating *2DS5* gene was found associated with risk for GC, and of note, a recent meta-analysis concluded that *2DS5* was associated with risk for colorectal cancer^29^; these results would suggest that *2DS5* may be associated with cancers in the gastrointestinal tract.

Concerning the inhibitory genes, our results were contrasting; whereas *2DL5* was associated with risk for GC, *3DL1* showed association with protection. Interestingly, a study in Chinese population found that the expression of *3DL1* in NK cells was significantly increased in patients with gastric, pancreatic and colorectal cancer, but was not correlated with disease progression^30^. The inhibitory genes have also been reported associated with other immune and infectious diseases; *3DL1* in combination with the HLA-B*57 allele showed a protective effect against progression to AIDS in Zambian patients^31^, and *2DL2* was reported associated with rheumatoid arthritis^28^.

In our population Bx genotype was frequent and associated with both gastric diseases, although the risk was stronger for NAG than for GC. In our patients Bx genotypes were a combination of A and B haplotypes, with very few B homozygotes, which is in agreement with studies in other human populations in America, including Amerindian groups^32^. This high frequency of Bx genotype may have resulted from the selection by the infectious and chronic diseases that have been prevalent in our population for many generations; although this selection process may have caused an increased risk for GC in the region.

Our work shows eight KIR genes associated with gastric diseases, five of them were associated with risk for GC (*2DS1*, *2DS3*, *2DS5*, *3DS1*, and *2DL5*) and belong to B haplotype, which is an activator haplotype. This unexpected association might partially be explained by the pathogenesis of GC^19^, which main risk factor is an infection. *H*. *pylori* infection is strongly pro-inflammatory and invariably causes a chronic, decades-long inflammation of the gastric mucosa^20^. In the context of a decades-long mucosal inflammation, NK cells may be constantly and chronically recruited and activated; until in some patients the regulation of this activation might be lost.^21^ Unregulated NK cell may help to increase inflammation leading to mucosal damage and development of precancerous lesions and eventually to GC^1,9,23,30^. On the other hand, activating KIR haplotypes would have opposite effects on distinct malignancies depending on whether inflammation is or is not a major component of tumor pathogenesis^33^. Although, it should be noted that an activator haplotype could also be expected to be associated with increased ability to eliminate tumors^1,34^. In fact, it was reported that patients with metastatic colorectal cancer had complete response to FOLFIRI (5-fluorouracil, leucovorin and irinotecan) treatment when B haplotype was present^35^. Interestingly, there was a strong association between the KIR B haplotype and p53 alteration in Basal cell carcinoma tumors, with a higher likelihood that KIR B carriers harbor abnormal p53^34^.

Concerning the distribution of A and B, we observed that A was more frequent in centromere and telomere of asymptomatic healthy adults, whereas B was more common in both, NAG and GC. The multivariate analysis confirmed a highly significant association of cA01|cB03 with GC, in fact this association (OR 36.39) was over 3 times higher than the association with age (OR 10.65 for >50 years old), which was usually the strongest factor. In addition, whereas tB01|tB01 was also a significant risk factor for GC, tA01|tA01 showed a significant association with protection. To our knowledge, there is no report describing the analysis of gene content in centromere and telomere regions and gastric cancer. Our analysis also confirms that the telomeric part of the KIR B genotype may have a role in the development of gastric diseases, particularly the cluster of genes *2DS1*, *2DS3*, *2DS5*, *3DS1* and *2DL5*, which showed a risk association with GC. In contrast, genes *2DS2* and *2DL2* were found as risk for NAG; these genes are located in the centromeric region of B haplotype, and have been reported with high linkage disequilibrium^16^. Thus, in our population the telomeric region of KIR B was more associated with GC, and the centromeric region with NAG. Within the B haplotype the telomeric region is more diverse, and probably the observed association with GC is due to an unbalanced response by the NK cell and a reduced ability to kill cancer cells. In contrast, the centromeric region is more conserve and the response of the NK cell is probably more balanced and efficient to fight cancer.

In order to determine the participation of B motifs in GC, we evaluated the B score in centromere and telomere, and the multivariate analysis confirmed its importance in GC, showing a trend following the course of the disease. A B-score of zero was more frequent in asymptomatics and strongly protective for GC (OR 0.23), and a score of 2 was associated with NAG, whereas a score of 3 increased the risk for GC almost 11 times (OR of 10.9). These results suggest that the exacerbated function of the B haplotype contributes to the damage of the gastric mucosa, favoring the development of GC. The B-score was previously evaluated in patients with acute myelogenous leukemia, where patients receiving transplant from donors with a B-score of 2 or greater showed a better protection from relapses, and an increased disease-free survival^18^; which would suggest that the exacerbated function of B results in an efficient response against leukemia.

On the other hand, the A genotype was associated with protection for GC, although only the *3DL1* gene showed a significant protection. This gene is within the telomeric region of A genotype and probably the observed association could be due to the strong linkage disequilibrium that *3DL1* has with the other genes of the A genotype.

It was recently reported that tumor-infiltrating NK cells were decreased in human GC; moreover, the production of IFNγ and TNFα by these cells was impaired by tumor-associated monocytes/macrophages via TGFβ1^36^. In contrast, patients with GC had a better survival when they presented higher concentrations of NK cells, an effect that was more evident in advanced stage cases^37^. Our work associates the risk to develop GC with the B KIR genotypes and the gene cluster included within the telomeric part. There is a need to better understand the functional role of the diversity in KIR genes content in GC, together with the participation of other factors involved in GC development, such as peptides derived from cancer that are presented by HLA class I molecules to KIR receptors^34^. Since HLA molecules are ligands of NK cells, they regulate the variation in immune responses to different antigens by selection and suppression/activation of NK cells, and have a relevant role in the combat against GC^9,38^. However, for the eight genes that presented association with NAG and GC in our study, only three ligands are known (*2DS1-C2*, *2DL2-C1* and *C2*, *3DL1-Bw4*)^38^. In addition, it is known that B allotypes can influence the binding with *3DL1*; thus, in the *Bw4* dimorphic position 80, isoluecine (HLA-B*51-*53, *57, *58, HLA-A*24) generally exhibit stronger inhibition than threonine (HLA-B*13, B*27, B*37, B*44)^33,39^. Besides, *3DL1* and the other KIR genes associated with GC reported in this study are expressed by CD56-dim NK cells, which migrate to acute inflammatory sites and display a higher cytotoxic activity than CD56-bright cells^40^, and the B haplotype could also influence the cytotoxic activity on tumor cells. It is necessary to further study the role of HLA-Cw and KIR gene alleles in gastric cancer surveillance since receptor-ligand combinations are important in the regulation of NK cell responses^38^.

Although one limitation of our study is the sample size, we were still able to identify a strongly significant risk association of a gene cluster located in telomeric region of B genotype with GC. We should consider that GC is a multifactorial disease and consequently a multivariate analysis is necessary to better understand the importance of KIR gene variants in GC. We acknowledge that whereas our work present evidences of a significant association of KIR gene variants with gastric pathology, this association is not probe of causality and further studies are now needed to show that unregulated NK cells in the stomach mucosa may lead to gastric pathology. In conclusion, we found that *2DS1*, *2DS3*, *2DS5*, *3DS1*, *2DL5*, Bx genotype, cBx-tBx, cA01|cB03, tA01|tB01, tB01|tB01 and B motifs were risk factors for GC. Mutated NK cells may contribute to GC development by increasing gastric mucosa inflammation, leading to constant tissue damage. The impact of the NK cell response on GC control might be determined in part by the genetic variation in KIR genes.

## Materials and Methods

### Study subjects

A total of 388 unrelated adults were recruited in this study, 146 healthy individuals (asymptomatic), 130 with non-atrophic gastritis (NAG) and 112 with GC. Patients with NAG were adults over 30 years old who were attended for symptoms at the gastroenterology service, whereas GC patients attended the oncology service for GC treatment; both groups attended the Instituto Mexicano del Seguro Social (IMSS) Medical Center in Mexico City. We selected NAG and GC patients without treatment of antibiotics, bismuth compounds, proton pump inhibitors and nonsteroidal anti-inflammatory drugs for at least two weeks prior to the study. GC patients without previous treatment for cancer were selected. Diagnosis was based on endoscopic examination and histopathology studies^41^. The individuals of the asymptomatic group were selected from healthy blood donors who attended the blood bank of the IMSS Medical Center, with an age over 30 years old and without any symptom or medication. To minimize the genomic diversity in different regions of the country of Mexico^42–45^, all groups of patients included in this study, patients and controls, received medical coverage from the same institute, IMSS at hospitals in the same city, Mexico City. Patients and controls were informed about the nature of the study and those willing to participate were asked to sign an informed consent letter. The study was approved by the ethics committee from the National Council for Research on Health, IMSS, Mexico and all procedures were performed in accordance with relevant guidelines and regulations.

### Collection of samples

For the NAG patients seven gastric biopsies were taken and processed for histology to study the presence of precancerous lesions and *H*. *pylori* infection. Biopsies were collected from both the lesser and the greater curvature, four from antrum and three from corpus. Mucosal inflammation was graded according to the Karttunen classification^46^, and only patients without precancerous lesions were included. In the case of GC patients, a tissue sample from the tumor lesion and a sample from adjacent non-cancerous tissue was also obtained and the lesion was classified according to the Karttunen classification^46^. A sample of 5 ml of peripheral blood was drawn from each patient, and each healthy volunteer, and mononuclear cells were purified by centrifugation through a Ficoll-Hypaque density gradient. DNA was isolated from these cells using the salting-out microtechnique^47^ and frozen at −70 °C until genotyping. The serum fraction was frozen at −20°C until tested.

### Definition of *H*. *pylori* infection

Serum samples were tested by ELISA to detect IgG antibodies against *H*. *pylori* whole-cell extract and against recombinant CagA protein, as previously described^48^. Infection was also diagnosed by histology in both antrum and corpus. The patient was considered infected with *H*. *pylori* when either tests, ELISA and/or histology, were positive, and non-infected when both tests were negative.

### Genotyping of KIR

The presence of each *KIR* gene was used to define the *KIR* gene content of patients. *KIR* genes were tested using a commercial kit (Invitrogene, Brown Deer, Wisconsin, USA) based on the technique of single specific primer-polymerase chain reaction (SSP-PCR), which can identify *2DL1*, *2DL2*, *2DL3*, *2DL4*, *2DL5A*, *2DL5B*, *2DS1*, *2DS2*, *2DS3*, *2DS4*, *2DS5*, *3DL1*, *3DL2*, *3DL3*, *3DS1*, *2DP1* and *3DP1* genes (including the variants of *2DS4* and *3DP1*). PCR reaction and cycling conditions were according the instructions recommended by the manufacturer.

### Statistical analysis

The gene, genotype, centromere-telomere gene content and B score frequencies in patients with NAG and GC were compared with the asymptomatic group. Chi-squared or Fisher’s exact test were used to test differences among groups, using the Epidat 3.1 Software; p values ≤ 0.05 were considered as significant^49^. The significance of association was assessed using odds ratios (OR) with confidence intervals (CI) of 95%^50^. OR values were corrected for gender and age using a logistic regression model. The analyses were performed using SPSS Statistics 22.0 (IBM SPSS Data Collection). The role of *H*. *pylori*, gender and age as variables influencing risk factor for GC was estimated in a multivariable logistic regression analysis.

### Data Availability

The datasets generated and analyzed during the current study are available from the corresponding authors on reasonable request.

## Electronic supplementary material

Distribution of KIR according to centromeric and telomeric gene content

Supplementary Dataset
