# Supplementary material for: Genotype B of Killer Cell Immunoglobulin-Like Receptor is Related with Gastric Cancer Lesions
Source: Sci Rep. 2018 Apr 17;8:6104. doi: 10.1038/s41598-018-24464-2 (PMC5904182; doi:10.1038/s41598-018-24464-2)
Supplement: Supplementary file 1 — Distribution of KIR according to centromeric and telomeric gene content [file 41598_2018_24464_MOESM1_ESM.docx]

**GENOTYPE B OF *KILLER CELL IMMUNOGLOBULIN-LIKE RECEPTOR* IS RELATED WITH GASTRIC CANCER LESIONS.**

Eric G. Hernandez^1^, Oswaldo Partida-Rodriguez^1^, Margarita Camorlinga-Ponce^2^, Miriam Nieves-Ramirez^1^, Irma Ramos-Vega^2^, Javier Torres^2*^, Martha Perez-Rodriguez^1*^

**Table 1S. Distribution of KIR according to centromeric and telomeric gene content**

|  | Asymptomatics | Non-atrophic gastritis | | Gastric cancer | |
| --- | --- | --- | --- | --- | --- |
|  | n = 146 ^a^ (%) | n = 130 ^a^ (%) | OR (95% CI) ^b^ | n = 112 ^a^ (%) | OR (95% CI) ^b^ |
| Centromere  cA01\|cA01 |  |  |  |  |  |
|  | 97 (66.4) | 50 (38.5) | 0.18 (0.087-0.36) | 64 (57.1) | NS |
| cA01\|cB01 | 10 (6.8) | 8 (6.2) | NS ^c^ | 9 (8.0) | NS |
| cA01\|cB02 | 35 (24.0) | 57 (43.8) | 4.18 (2.08-8.38) | 16 (14.3) | NS |
| cA01\|cB03 | 1 (0.7) | 8 (6.2) | 21.82 (1.36-349.76) | 12 (10.7) | 143.85 (8.65-2391.42) |
| cA01\|cB0X | 0 | 3 (2.3) | NS | 3 (2.7) | NS |
| cA0X\|cB02 | 1 (0.7) | 2 (1.5) | NS | 4 (3.6) | NS |
| cB01\|cB01 | 1 (0.7) | 1 (0.8) | NS | 4 (3.6) | NS |
| cB02\|cB02 | 1 (0.7) | 1 (0.8) | NS | 0 | NS |
| Telomere  tA01\|tA01 |  |  |  |  |  |
|  | 89 (61.0) | 14 (10.8) | 0.067 (0.03-0.16) | 27 (24.1) | 0.16 (0.06-0.40) |
| tA01\|tB01 | 43 (29.5) | 66 (50.8) | 2.99 (1.56-5.72) | 59 (52.7) | 2.2 (0.96-5.03) ^c^ |
| tA01\|tB0X | 3 (2.1) | 50 (38.5) | 37.10 (7.47-184.23) | 13 (11.6) | 7.62 (0.65-89.68) ^c^ |
| tA0X\|tB01 | 6 (4.1) | 0 | NS | 0 | NS |
| tB01\|tB01 | 5 (3.4) | 0 | NS | 13 (11.6) | 65.11 (4.11-1030.39) |

^a^ n= number of subjects; ^b^ Comparisons were made using the asymptomatic group as the reference group, *pc*<0.05 and OR (95% C.I.) adjusted by age and gender. ^c^ *pc*>0.05. ^c^ NS = not significant. The number was according gene content^15-17^. c = centromere. t = telomere. 0X = the number had not been assigned so far.
